# Role of Scavenger Receptors in Glia-Mediated Neuroinflammatory Response Associated with Alzheimer's Disease

**DOI:** 10.1155/2013/895651

**Published:** 2013-05-07

**Authors:** Francisca Cornejo, Rommy von Bernhardi

**Affiliations:** Laboratorio de Neurociencias, Departamento de Neurología, Escuela de Medicina, Pontificia Universidad Católica de Chile, Marcoleta, 391 Santiago, Chile

## Abstract

It is widely accepted that cells serving immune functions in the brain, namely, microglia and astrocytes, are important mediators of pathological phenomena observed in Alzheimer's disease. However, it is unknown how these cells initiate the response that results in cognitive impairment and neuronal degeneration. Here, we review the participation of the immune response mediated by glial cells in Alzheimer's disease and the role played by scavenger receptors in the development of this pathology, focusing on the relevance of class A scavenger receptor (SR-A) for A**β** clearance and inflammatory activation of glial cell, and as a potential target for Alzheimer's disease therapy.

## 1. Introduction

Alzheimer's disease (AD) is the most prevalent form of dementia, being usually diagnosed in people over 65 years, although the less prevalent early-onset forms of AD develop at earlier ages [1]. AD shows an increasing prevalence mainly due to the population aging. Dementia prevalence rises exponentially, doubling the rate of AD every 5 years after the age of 60 with a 15-fold prevalence increment from-age-60 to 85 years [2].

The pathophysiology of this dementia is characterized by the extracellular accumulation of amyloid-*β* (A*β*) and the intracellular formation of neurofibrillary tangles of the Tau protein in neurons, in association with neuronal dysfunction and cell death in some brain areas as the hippocampus. Although A*β* has been considered to be the main agent implicated in AD pathogenesis, it is still uncertain if A*β* plaques are causative for AD or a consequence of its pathological changes.

There are several hypotheses that attempt to explain the origin of AD [3], although the most popular is still the “A*β* cascade hypothesis” [4], which considers A*β* as the key pathogenic factor. The “inflammation hypothesis” [5] and the “glial dysfunction hypothesis” [6] have lately gained increased support. With some differences, both of them state that A*β* accumulation is a consequence of the dysregulated activation of glial cells, which in turn induce an inflammatory response, alter their A*β*-clearance activity, and mediate the neurotoxic effects of A*β* [7, 8]. 

## 2. Glial Dysfunction Hypothesis

It is widely known that aging, the most robust risk factor for AD, is also strongly associated with a progressive increment on the inflammatory state of the organism. Inflammation induces a large amount of cell changes at multiple levels, including microglial cells [9], and, as it will be discussed in the next section, microglial cells also become more neurotoxic in response to inflammatory states [10]. Whereas the inflammation hypothesis considers that hyperreactive microglia is the major contributor to the adverse events associated with AD, the glial dysfunction hypothesis suggests that impairment of normal glial functions, meaning qualitative changes, and not only quantitative changes on microglial cell activation, are responsible for the synaptic dysfunction and the neurodegenerative process observed in AD [6]. 

As it will be further discussed, glia are the scavenger cells of the brain. By having a reduced capability to clear A*β* [11], A*β* accumulates and microglial cells become activated and create a cytotoxic environment that induce a vicious circle that potentiates a neuroinflammatory state and neurotoxicity [12, 13]. Because the impairment of A*β* clearance induce the accumulation of the peptide even if there are no changes in A*β* production, this hypothesis states that A*β* accumulation would be a consequence and not a cause of the pathogenic changes leading to AD [14, 15]. 

## 3. Neuroinflammatory Response in AD 

### 3.1. Microglial Cell Response

There is robust evidence showing high levels of inflammatory mediators in the brain of AD patients. Around senile plaques, a strong presence of TNF-*α*, IL-1*β*, IL-6, monocyte chemoattractant protein-1 (MCP-1), complement proteins as C1q, C1r, C2, C3, C4, C5, C6, C7, C8, and C9, C-reactive protein, and class II major histocompatibility complex antigen HLA-DR is observed [16–20]. When the effect of inflammatory cytokines over the production of A*β* was evaluated, it was demonstrated that exposure to cytokines such as IL-6 and IL-1, increase neuronal amyloid precursor protein (APP) mRNA expression [21]. In addition, glial cultures obtained from rapid brain autopsies of AD patients stimulated with A*β* show an increased release of prointerleukin-1*β* (pro-IL-1*β*), IL-6, IL-8, TNF-*β*, MCP-1, macrophage inflammatory peptide-1*α* (MIP-1*α*), and macrophage colony-stimulating factor (M-CSF) [22], corroborating the inflammatory activation of glial cells as part of the physiopathology of AD. Despite of this, *in vitro* studies have demonstrated that A*β* potentiates inflammatory activation of microglia [23], with different forms of A*β* showing distinct patterns of cytokine release; for instance, soluble forms of A*β*_1–40_ stimulate specifically the release of IL-1*β*, while A*β*_1–42_ induces the release of IL-1*α* and IFN-*γ* [24].

Also, immunohistochemical studies of the brain of AD patients have shown the presence of reactive microglia closely associated with senile plaques [20, 25]. The exposure of microglia to a soluble form of APP (sAPP) induces an increase of activation markers in microglia and enhances their production of neurotoxins [26]. More specifically, A*β* stimulates the NF*κ*B-dependent pathway [27], which in turn induces the cytokines production by microglia and initiates the neurotoxic effects mediated by these cells [17]. 

Using mouse models of AD, Simard et al. showed that amyloid plaques are capable of chemoattracting immune cells from the bone marrow into the brain parenchyma, which adopt a microglial cell phenotype in the brain and suggested that these immigrant cells are the main responsible for A*β* plaque clearance [28]. Microglial cell-associated A*β* clearance was originally shown by incubating murine microglia with fluorescent-labeled A*β* [11]. The study also established the participation of scavenger receptors (SRs) in this process by demonstrating that coincubation with an excess of SRs ligands blocked the phagocytosis of A*β*. Also, the use of Chinese hamster ovary (CHO) cells transfected with scavenger receptors of class A (SR-A) and class B (SR-B) significantly potentiated the uptake of A*β* [11], situating SRs as the principal receptors responsible for senile plaques clearance. 

Although the mechanistic factor involved in the association between AD and aging is still an unsolved question, there is evidence pointing out to microglial aging as well as other age-related changes as responsible for this correlation [9]. Studies of adult cortical cells have shown a reduced capacity of aged microglia to phagocytose A*β* [29]. This decrease in phagocytic activity was mainly favored by a proinflammatory state [30]. In addition, it has been shown that A*β* has cytotoxic effects only in aged individuals, with no A*β*-induced neurodegeneration observed in the brain of young animals [31].

All these microglial-mediated effects observed in AD are contrasted to the normally neuroprotective role of microglia, which involves the phagocytic capacity responsible for brain surveillance from infection and physical injuries, the supportive role implicated in neuronal survival by secreting nerve growth factors, and the contribution in creating a microenvironment for central nervous system (CNS) regeneration [32, 33]. These changes in normal microglial cell functions are usually explained as a switch in the inflammatory state of the cell: during aging (and possibly also in AD), microglia change from an M2 activation state, characterized as an anti-inflammatory phenotype associated with wound healing, to an M1 state, which is a proinflammatory activation state related to the production of inflammatory cytokines, chemokines, and reactive intermediates [34].

This evidence leads us to propose that aging, which is commonly associated with a progressive inflammatory state of the brain, can be one of the most important causes of the defective clearance of neurotoxic A*β*, which in turn favors the overstimulation of the immune response, creating a positive feedback that leads to neuronal dysfunction, neurodegeneration, and the progressive development of neurodegenerative diseases like AD. 

### 3.2. Astrocytes Response

As for microglia, astrocytes have also been observed in close proximity to senile plaques of AD patient brains [35]. Moreover, A*β* is capable of stimulating the production of MCP-1 in astrocytes [36], having an important role in chemotaxis for attracting immune cells to the senile plaque. 

In AD patient brains, an upregulation of IFN-*γ* receptor (IFNGR) on activated astrocytes has been observed, where treatment with INF-*γ* resulted in reduced cell viability [37], suggesting that activated astrocytes can become neurotoxic at least under certain conditions of inflammatory stimulation.

Under physiological conditions, astrocytes are the cells that maintain the brain integrity: they provide metabolic support for neurons; are capable of sensing and modulate the neuronal environment; regulate the synaptic levels of glutamate, ion concentrations, and the acid-base balance; synthesize and release antioxidant molecules; participate in the formation of the blood brain barrier; and function as immune competent cells by acting in the clearance of cell debris and as antigen presenting cells [38]. However, the effects mediated by astrocytes in AD are mainly harmful [39], which reveals the dual properties of astrocytes depending on the cellular context in which they are immersed. 

In AD animal models, it has been demonstrated that astrocytes surrounding A*β* plaques are immunopositive for IL-6 [40]. In the same line of evidence, *in vitro* experiments show that A*β* exposure has differential immune effects in astrocytes depending on the peptide conformation: oligomeric A*β* induce transient high levels of IL-1*β* with a fast decrease, increasing nitric oxide (NO) production, inducible nitric oxide synthase (iNOS) and TNF-*α* expression, consistent with an early inflammatory response, while fibrillar A*β* induces persistent increased levels of IL-1*β* that remains over time, corresponding to a more chronic response [41]. The release of IL-1*β* by A*β*-stimulated astrocytes promotes the release of IL-1*β*, IL-6, and TNF-*α* by microvascular endothelial cells, suggesting that astrocytes-cytokine release also plays a role in neuroinflammation and endothelial response that contribute to AD progression [42].

*In vitro* studies have also shown that astrocytes exposed to A*β* present sporadic cytoplasmic calcium signals that correlate with the death of adjacent neurons, an effect that is, abolished by pretreating cells with heavy-metal chelators [43]. This effect suggests a neurotoxic effect mediated by intracellular calcium increase in astrocytes induced by A*β*. In addition, it was found that astrocytes exposed to A*β* have an increased glucose uptake and hydrogen peroxide production with no changes in intracellular antioxidants, both effects mediated by activation of the PI3K pathway [44], indicating that A*β* induces alterations of astrocytes metabolism [45] that could result in increased cytotoxicity.

Using specific deletion of the immune calcineurin/nuclear factor of activated T-cell (NFAT) pathways in astrocytes, which mediates biochemical pathways leading to astrocytes activation, it has been shown in AD animal models that activated astrocytes are responsible for cognitive and synaptic function impairment mediated by amyloid depositions [46], confirming a deleterious role of activated astrocytes in AD.

Nevertheless, even though activated astrocytes appear to have a deleterious role in AD progression, there is also evidence showing that they are capable of ameliorating the cytotoxic effects of activated microglia in culture. Conditioned media from microglia exposed to A*β* induce apoptosis in hippocampal cells, but this effect is not observed when the media is obtained from mixed glial cultures exposed to A*β*. Many of the inflammatory activation changes of microglial cell induced by A*β* are attenuated in the presence of astrocytes [47]. Moreover, astrocytes activation mediated by LPS and IFN-*γ* induce the secretion of TGF-*β*, a neuroprotective cytokine, which was capable of reducing apoptosis of hippocampal cells induced by A*β* [48], suggesting that astrocytes have a pivotal role in the modulation of AD inflammation. 

Although the mechanisms that mediate astrocytes changes induced by A*β* are poorly understood, there is evidence showing that astrocytes are capable of phagocytosing A*β* and that they interact with A*β* through the SRs [23], specifically pointing out to SR-A as responsible for this interaction [35, 44, 49], which allows one to infer that, as in microglia, the phagocytic activity by astrocytes could be impaired with aging. Furthermore, given the high number of astrocytes in the brain parenchyma, even if the phagocytic activity of astrocytes appears to be less robust than that of microglia, changes in phagocytosis can be highly relevant for a decreased A*β* clearance capability, as well as impairment of the regulation of microglial cells [9].

### 3.3. Scavenger Receptors in AD

Cells mediating the immune response interact with multiple environmental compounds, and depending on receptors present on their surface, their response to those signals could be pro- or anti-inflammatory. Many pattern recognition receptors (PRR) have been described, like Toll-like receptors (TLR) and Nod-like receptors (NLR) that trigger the activation of specific inflammatory pathways according to the ligand they bind [50]. In addition, many immune cells are able to phagocytose diverse compounds because of the presence of receptors that uptake various ligands such as cell debris and allow their removal through the lysosomal pathway [51]. Many of these receptors belong to the scavenger receptors (SRs) family, a term that was first coined in 1979 to define high-affinity binding sites for acetylated low-density lipoproteins (acLDL) on macrophages [52]. These receptors share the capability of binding polyanionic ligands without differentiating exogenous ligands like those from pathogens and endogenous ligands, which have importance in the host defense response.

Whereas participation of SRs has been widely described in atherosclerosis, their role in AD-associated immune response remains poorly understood. However, it has been shown that SRs have an important role in the clearance of A*β*, and that the expression of these receptors decreases in aging brains of animal models of AD [53], situating SRs as important mediators of AD progression.

**Figure 1:**
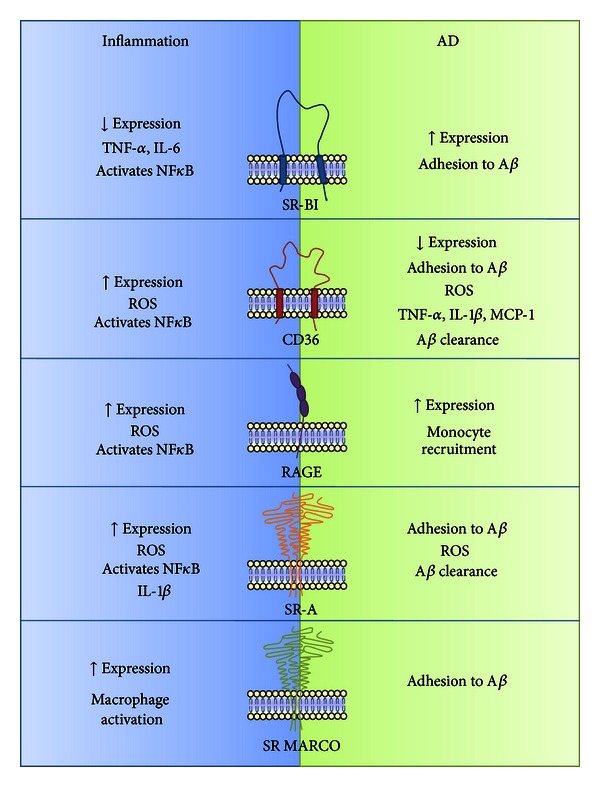
Summary of the main characteristics and functions played by SRs in inflammation and AD. The left panel shows changes induced by the inflammatory activation of SRs reviewed in the text. The right panel resumes the principal alterations induced by the activation of SRs mediated by the amyloid peptide.

Until now 6 families of SRs have been described, named from SR-A to SR-F, but there are still 3 SRs that remain unclassified, which are RAGE, CD136, and SR-PSOX. It is unknown if the last two receptors are associated with the pathophysiology of AD, even though both are expressed in the CNS [54]. In this section we will discuss the main SRs that appear to be involved in AD and their role in glial cell inflammatory activation (Figure 1). 

### 3.4. SR-BI

The principal ligand of SR-BI is HDL [55], which has an important role in lipid and cholesterol mobilization in the “reverse cholesterol transport” [56]. Because of the delivery of cholesterol from peripheral tissue occurs in SR-BI-expressing cells participating in lipid metabolism, the main expression of this receptor can be found in the liver and in steroidogenic tissues. Nevertheless, SR-BI can be also found in the brain parenchyma, especially on glial cells [49] and cerebral arterial smooth muscle [57]. 

It is believed that SR-BI has a role in host defense [58] because of the upregulation of its expression during phagocytic and dendritic differentiation of monocytes and because of the suppression of its expression in monocytes and macrophages, exposed to proinflammatory stimuli [59]. Moreover, SR-BI-null mice have a 100% fatality induced by sepsis with increased levels of inflammatory cytokines released by macrophages [60], while overexpression of SR-BI attenuated the inflammatory response in these cells [61], suggesting a protective role of SR-BI through the modulation of the inflammatory response of macrophages.

In astrocytes, the main role of SR-BI appears to be associated with clearance of apoptotic cells [62]. However, it has also been reported that SR-BI is involved in the adhesion of microglia to A*β* plaques [63]. Studies have shown that animal models for A*β* accumulation have an increased expression of SR-BI in the brain, and partial or total deletion of SR-BI gene in those animals enhances A*β* deposition associated with an impaired response of perivascular macrophages to A*β* [64]. However, if SR-BI has a direct role in A*β* clearance and if the interaction between A*β* and SR-BI activates specific signaling pathways remain as unanswered questions. 

### 3.5. CD36

CD36, another member of the SR-B family, was initially described in adipocytes and myocardium, where it participates in the transport of long-chain fatty acids (LCFA) [65]. The deficiency of CD36 leads to an increase in plasma levels of free fatty acids and triacylglycerol, with an abnormal myocardial LCFA uptake [66]. CD36 is also expressed in immune cells, being associated with the clearance of apoptotic neutrophils by macrophages [67]. In the brain, the expression of CD36 has been reported in capillary endothelium [68], astrocytes [69], and microglial cells [70], in which this receptor has been associated with the regulation of cell migration [71].

CD36 appears to be involved in pathologies such as brain ischemia, where CD36 expression is increased mostly in cells expressing the microglia/macrophage marker CD11b. CD36-null mice have reduced infarct size after ischemia, improved neurological function, and, less ischemia-induced reactive oxygen species (ROS) levels than wild-type animals [72]. CD36-null mice also have an attenuated postischemic activation of NF*κ*B, suppressed glial activation [73], and an impaired astrocytes proliferation [69], all of which situate CD36 as an important mediator for brain inflammatory events. 

Besides brain ischemia, Coraci et al. detected reduced levels of CD36 in microglia obtained from patients with AD, multiple sclerosis, and Parkinson's disease [70]. Specifically, in AD, CD36 appears to have a major role in the binding of cells of the monocyte/macrophage lineage to A*β*, which activates a signaling pathway associated with production of ROS and cytokines [74, 75]. In CD36-null mice, microglia and macrophages have reduced secretion of cytokines, chemokines, and ROS in response to treatment with A*β*, in addition to showing a decreased macrophage and microglial recruitment into the brain [76].

In contrast to the reports by Coraci et al. [70], Ricciarelli et al. reported high expression of CD36 in the cerebral cortex of AD patients and in normal subjects with diffuse amyloid plaques, compared with the amyloid-free brains of control individuals. Also, by using cells *in vitro*, they demonstrated that A*β*-induced CD36 overexpression in neurons was associated with an increase in nitrated proteins [77]. Nonetheless, CD36 expression by leukocytes is significantly reduced in AD patients, a phenomenon already observed at early preclinical stages as mild cognitive impairment [78].

Otherwise, in animal models of AD, it has been shown that old mice had a decreased expression of CD36 associated with an increased secretion of IL-1*β* and TNF-*α* [53], and an increased vascular amyloid deposition mediated by CD36 [79].

The ability of CD36 to participate in the clearance of A*β* has been demonstrated by Shimizu et al., who by using CHO cells overexpressing CD36, showed a dose-dependent degradation of labeled A*β*, during treatment with an anti-CD36 antibody blocked A*β* degradation [80]. In addition, astrocytes-mediated A*β* clearance is also attenuated with neutralizing antibodies against CD36 [81].

Although there are only few studies that associate CD36 with A*β* clearance, it appears that this receptor is mostly associated with neurovascular dysfunction observed in AD. In animal models of AD, deficiency of CD36 prevents cerebrovascular effects and oxidative stress elicited by A*β* [79, 82], suggesting that CD36 could be a therapeutical target mainly for the treatment of neurovascular dysfunction in AD patients.

### 3.6. RAGE

RAGE is a member of the immunoglobulin family and a cell surface receptor for advanced glycation endproducts (AGEs), which accumulate mainly in vascular tissues in aged individuals [83]. RAGE protein expression can be found in vasculature, endothelium, smooth muscle, mononuclear cells, cardiac myocytes, and neural tissue [84]. 

The interaction of RAGE with the ligand amphoterin, a polypeptide associated with the growth of cortical neurons derived from the developing brain, has been linked to cancer as the colocalization of both molecules has been shown to contribute to cellular migration and tumor invasion. Blockade of this interaction leads to suppression of the activation of intracellular pathways linked to tumor proliferation [85].

Interaction of AGEs with RAGE expressed by endothelial cells has been usually related to vascular dysfunction, mainly because of ROS induced by AGEs. This oxidative stress results in the activation of NF*κ*B pathway [86], an affect that has been also observed in inflamed gut biopsies with a significant upregulation of RAGE [87], situating RAGE as an important inflammatory mediator in AGEs mediated lesions. 

In relation to AD, the interaction of A*β* with RAGE expressed by endothelial cells of the brain favors the transendothelial migration of monocytes from peripheral blood into the brain, indicating an important role of RAGE in AD-related vascular disorders [88]. Also, an increased expression of RAGE by neurons in the brain of AD patients has been observed. Murine models of AD with overexpression of RAGE have an exacerbated impairment in spatial learning/memory and altered activation of synaptic plasticity markers [89], where the synaptic depression and LTD impairment induced by A*β* could be rescued by functional suppression of RAGE activity in microglia [90]. However, Vodopivec et al. have shown that the absence of RAGE in animal models of AD does not ameliorate their cognitive deterioration, A*β* accumulation, or microglial activation [91], suggesting that RAGE would have only a secondary role for the impairments observed in AD.

### 3.7. SR-A

Scavenger receptor class A (SR-A) is a homotrimeric transmembrane glycoprotein containing extracellular C-terminal cysteine-rich domains, that was initially implicated in the development of atherosclerosis, because of its colocalization with macrophages of lipid-rich atherosclerotic lesions [92, 93]. The expression of this receptor has been detected in many tissues, including liver, placenta and brain [94]. When discovered, the first function described for SR-A was to provide adhesiveness to monocytes and macrophages to glycated collagen-IV-coated surfaces, and to mediate the endocytosis of acLDL [95]. Also, given the fact that macrophage SRs are involved in the binding and internalization of LPS, which is part of Gram-negative bacteria [96], Thomas et al. showed that SR-A-deficient mice were more susceptible to infection with pathogens, with an impaired ability to clear bacterial infection, confirming what was shown by Suzuki et al., providing the first insight on the importance of SR-A in host defense [97, 98]. Additionally, other researchers observed that these animals showed a reduced expression of IL-1*β*, which is associated with a reduced mortality in response to LPS [99], showing that SR-A has a role in the macrophage activation induced by endotoxin shock [100–104].

In addition, SR-A also plays a role in macrophage engulfment of apoptotic thymocytes [105, 106], and in atherosclerosis, observing that disruption of the SR-A gene results in a reduction in the size of atherosclerotic lesions [98]. Moreover, in left ventricular remodeling after myocardial infarction, SR-A has a role in attenuating cardiac remodeling by suppressing macrophage polarization toward a biased M1 phenotype, reducing the release of proinflammatory cytokines [107].

It is important that in contrast to most SRs, SR-A expression is not downregulated by chronic exposure to endogen ligands such as acLDL. On the opposite, SR-A expression can be reversibly increased by incubating macrophages with SR-A ligands [108]. In addition, binding of a ligand to SR-A activates PI3K recruits more receptors to the membrane surface [109], all of which are key functional characteristic to consider when SR-A is seen as a therapeutic target. 

In the normal brain, SR-A is expressed by microglia, perivascular cells, microvessels, stromal, epiplexus, and meningeal macrophages [110–114]. Furthermore, our group was the first to describe the expression of SR-A by astrocytes, showing that exposure to SR-A ligands activated MAPKs and NF*κ*B signaling pathways and increased the production of IL-1*β* and NO by astrocytes [115]. 

In an animal model of cerebral ischemic injury, SR-A is upregulated in the brain, an effect that correlates with increased levels of proinflammatory markers in microglia/macrophages, and an increased activation of NF*κ*B; whereas SR-A-deficient mice show a reduced infarct size and improved neuronal function, suggesting the participation of SR-A into the M1 microglia/macrophage polarization [116–118].

In AD, SR-A has been observed in close association with senile plaques [111], presenting microglia positive for the receptor [114, 119]. SR-A has been related to rodent microglia and monocyte adhesion to A*β* coated surfaces, leading to the production of ROS [120] and to A*β* internalization mediated by endosomes in microglia [57, 121, 122]. More specifically, A*β* internalized through SR-A is trafficked toward lysosomes inside the microglia and degraded by cathepsin B [123]. To our knowledge, SR-A appears to be the most important scavenger receptor of the brain participating in A*β* clearance. For this reason, SR-A could be a potential therapeutical target in the treatment of AD.

### 3.8. SR MARCO

The macrophage receptor with collagenous structure (MARCO) is a member of the SRs class A family, localized mainly in macrophages of the spleen and lymph nodes [124], acting in the binding of bacterial antigens and phagocytosis, and having an important role in host defense [125]. In the presence of pro-inflammatory stimuli like LPS, SR MARCO expression is upregulated even in macrophages from liver and lungs, where normally it is not expressed [126]. It is believed that SR MARCO has a direct effect in the morphology of activated macrophages, which is necessary for trapping pathogens, by mediating the formation of lamellipodia-like structures and dendritic processes [127]. In fact, SR MARCO expression is essential for dendritic cells to acquire a mature phenotype [128].

In the CNS, SR MARCO is present in microglia and astrocytes; SR MARCO expression in microglia is associated with a decrease of the antigen internalization capacity [128], while for both astrocytes and microglial cells it is believed that SR MARCO participates in their adhesion to A*β* [49].

### 3.9. Other SRs in AD

There are other SRs that have been involved in the signaling mediated by A*β*, which we will briefly mention because of the scarce evidence that relates them directly to the pathophysiology of AD.

#### 3.9.1. CD68

This receptor, also known as macrosialin, is a member of the lysosomal-associated membrane protein (LAMP) family, which is expressed in macrophages, osteoclasts, dendritic cells, and microglia, where its principal role is to bind and uptake oxidized lipoproteins and apoptotic cells [129]. Although there are no studies involving CD68 in AD, Argiles et al. showed that patients with haemodialysis-associated amyloidosis show an upregulated expression of CD68 by macrophages [130].

#### 3.9.2. OLR1

The oxidized LDL receptor 1 (OLR1) binds LDL, being an important SR associated with atherosclerosis, is mainly expressed by endothelial cells and monocytes, with minor expression by macrophages [131]. Even though it is unknown if OLR1 is expressed by microglia, and if the receptor has a direct role in the immune response, it has been shown that OLR1 is associated with A*β* transport across the blood brain barrier [132, 133].

#### 3.9.3. MEGF10

Multiple EGF-like domains 10 (MEGF10) is a type 1 transmembrane protein containing 17 EGF-like domains in the extracellular portion [134] and is mainly expressed in the brain, and it has been shown to be implicated in the uptake of A*β* mediated by the lipid rafts endocytosis pathway [135]. Nevertheless, MEGF10 expression has not been observed in glial cells; therefore, the mechanism by which this receptor could participate in A*β* clearance is unknown. 

## 4. Conclusion

When AD is visualized as a pathology caused by a dysfunction of glial cells, which compromise several of their protective functions, including the phagocytosis of A*β*, and favors potentially deleterious effects, as those observed in dysregulation of the inflammatory regulation, an objective target to generate potential treatments should be the recovery of the protective activation of glia, characteristics that appear to be lost in association with aging and chronic inflammatory activation. Most of SRs found in glia appear to be potentially involved in phagocytosis and inflammatory activation of glial cells. However, they have been poorly studied in terms of their interaction with the A*β* peptide. 

As it was shown by Hickman et al., the age progression associated with AD reduces the expression of SR-A in older individuals, an effect that is, also induced by treating microglial cells with pro-inflammatory cytokines [53]. This robust evidence situates SR-A as one of the main receptors involved in the impaired clearance of A*β* observed in AD and also could be the link between AD and the inflammatory state related to aging, suggesting that SR-A could be an interesting therapeutic target for AD.

## References

[1] Lobo A, Launer LJ, Fratiglioni L (2000). Prevalence of dementia and major subtypes in Europe: a collaborative study of population-based cohorts. *Neurology*.

[2] Mayeux R, Stern Y (2012). Epidemiology of Alzheimer disease. *Cold Spring Harbor Perspectives in Medicine*.

[3] von Bernhardi R, Eugenin J (2012). Alzheimer's disease: redox dysregulation as a common denominator for diverse pathogenic mechanisms. *Antioxidants and Redox Signaling*.

[4] Hardy JA, Higgins GA (1992). Alzheimer’s disease: the amyloid cascade hypothesis. *Science*.

[5] Aisen PS, Davis KL (1994). Inflammatory mechanisms in Alzheimer’s disease: implications for therapy. *American Journal of Psychiatry*.

[6] von Bernhardi R (2007). Glial cell dysregulation: a new perspective on Alzheimer disease. *Neurotoxicity Research*.

[7] Qiu C, Kivipelto M, von Strauss E (2009). Epidemiology of Alzheimer’s disease: occurrence, determinants, and strategies toward intervention. *Dialogues in Clinical Neuroscience*.

[8] Mattsson MO, Simko M (2012). Is there a relation between extremely low frequency magnetic field exposure, inflammation and neurodegenerative diseases? A review of in vivo and in vitro experimental evidence. *Toxicology*.

[9] von Bernhardi R, Tichauer JE, Eugenín J (2010). Aging-dependent changes of microGlial cells and their relevance for neurodegenerative disorders. *Journal of Neurochemistry*.

[10] Solito E, Sastre M (2012). MicroGlia function in Alzheimer's disease. *Frontiers in Pharmacology*.

[11] Paresce DM, Ghosh RN, Maxfield FR (1996). MicroGlial cells internalize aggregates of the Alzheimer’s disease amyloid *β*-protein via a scavenger receptor. *Neuron*.

[12] von Bernhardi R, Ramírez G, Toro R, Eugenín J (2007). Pro-inflammatory conditions promote neuronal damage mediated by Amyloid Precursor Protein and decrease its phagocytosis and degradation by microGlial cells in culture. *Neurobiology of Disease*.

[13] Ramírez G, Rey S, von Bernhardi R (2008). Proinflammatory stimuli are needed for induction of microGlial cell-mediated A*β*PP_{244-C} and A*β*-neurotoxicity in hippocampal cultures. *Journal of Alzheimer’s Disease*.

[14] Harry GJ, Kraft AD (2008). Neuroinflammation and microGlia: considerations and approaches for neurotoxicity assessment. *Expert Opinion on Drug Metabolism and Toxicology*.

[15] Luo XG, Ding JQ, Chen SD (2010). MicroGlia in the aging brain: relevance to neurodegeneration. *Molecular Neurodegeneration*.

[16] Rogers J, Luber-Narod J, Styren SD, Civin WH (1988). Expression of immune system-associated antigens by cells of the human central nervous system: relationship to the pathology of Alzheimer’s disease. *Neurobiology of Aging*.

[17] Combs CK, Colleen Karlo J, Kao SC, Landreth GE (2001). *β*-amyloid stimulation of microGlia anti monocytes results in TNF*α*-dependent expression of inducible nitric oxide synthase and neuronal apoptosis. *The Journal of Neuroscience*.

[18] Yasojima K, Schwab C, McGeer EG, McGeer PL (1999). Up-regulated production and activation of the complement system in Alzheimer’s disease brain. *American Journal of Pathology*.

[19] Strauss S, Bauer J, Ganter U, Jonas U, Berger M, Volk B (1992). Detection of interleukin-6 and *α*2-macroglobulin immunoreactivity in cortex and hippocampus of Alzheimer’s disease patients. *Laboratory Investigation*.

[20] Ishizuka K, Kimura T, Igata-Yi R, Katsuragi S, Takamatsu J, Miyakawa T (1997). Identification of monocyte chemoattractant protein-1 in senile plaques and reactive microGlia of Alzheimer’s disease. *Psychiatry and Clinical Neurosciences*.

[21] Del Bo R, Angeretti N, Lucca E, de Simoni MG, Forloni G (1995). Reciprocal control of inflammatory cytokines, IL-1 and IL-6, *β*-amyloid production in cultures. *Neuroscience Letters*.

[22] Lue LF, Rydel R, Brigham EF (2001). Inflammatory repertoire of Alzheimer’s disease and nondemented elderly microGlia in vitro. *Glia*.

[23] Murgas P, Godoy B, von Bernhardi R (2012). Abeta potentiates inflammatory activation of Glial cells induced by scavenger receptor ligands and inflammatory mediators in culture. *Neurotoxicity Research*.

[24] Lindberg C, Selenica MLB, Westlind-Danielsson A, Schultzberg M (2005). *β*-amyloid protein structure determines the nature of cytokine release from rat microGlia. *Journal of Molecular Neuroscience*.

[25] Dickson DW, Farlo J, Davies P, Crystal H, Fuld P, Yen SHC (1988). Alzheimer’s disease. A double-labeling immunohistochemical study of senile plaques. *American Journal of Pathology*.

[26] Barger SW, Harmon AD (1997). MicroGlial activation by alzhelmer amyloid precursor protein and modulation by apolipoprotein E. *Nature*.

[27] Flores B, von Bernhardi R (2012). Transforming growth factor beta1 modulates amyloid beta-induced Glial activation through the Smad3-dependent induction of MAPK phosphatase-1. *Journal of Alzheimer's Disease*.

[28] Simard AR, Soulet D, Gowing G, Julien JP, Rivest S (2006). Bone marrow-derived microGlia play a critical role in restricting senile plaque formation in Alzheimer’s disease. *Neuron*.

[29] Floden AM, Combs CK (2006). *β*-amyloid stimulates murine postnatal and adult microGlia cultures in a unique manner. *The Journal of Neuroscience*.

[30] von Bernhardi R, Tichauer J, Eugenin-von Bernhardi L (2011). Proliferating culture of aged microGlia for the study of neurodegenerative diseases. *Journal of Neuroscience Methods*.

[31] Geula C, Wu CK, Saroff D, Lorenzo A, Yuan M, Yankner BA (1998). Aging renders the brain vulnerable to 26 *β*-protein neurotoxicity. *Nature Medicine*.

[32] Barron KD (1995). The microGlial cell. A historical review. *Journal of the Neurological Sciences*.

[33] Napoli I, Neumann H (2010). Protective effects of microGlia in multiple sclerosis. *Experimental Neurology*.

[34] Boche D, Perry VH, Nicoll JA (2013). Review: activation patterns of microGlia and their identification in the human brain. *Neuropathology and Applied Neurobiology*.

[35] Nagele RG, D’Andrea MR, Lee H, Venkataraman V, Wang HY (2003). Astrocytes accumulate A*β*42 and give rise to astrocytic amyloid plaques in Alzheimer disease brains. *Brain Research*.

[36] Smits HA, Rijsmus A, Van Loon JH (2002). Amyloid-*β*-induced chemokine production in primary human macrophages and astrocytes. *Journal of Neuroimmunology*.

[37] Hashioka S, Klegeris A, Schwab C, McGeer PL (2009). Interferon-*γ*-dependent cytotoxic activation of human astrocytes and astrocytoma cells. *Neurobiology of Aging*.

[38] Fuller S, Steele M, Münch G (2010). Activated astroGlia during chronic inflammation in Alzheimer’s disease—do they neglect their neurosupportive roles?. *Mutation Research*.

[39] Heneka MT, O’Banion MK, Terwel D, Kummer MP (2010). Neuroinflammatory processes in Alzheimer’s disease. *Journal of Neural Transmission*.

[40] Benzing WC, Wujek JR, Ward EK (1999). Evidence for Glial-mediated inflammation in aged APP(SW) transgenic mice. *Neurobiology of Aging*.

[41] White JA, Manelli AM, Holmberg KH, van Eldik LJ, LaDu MJ (2005). Differential effects of oligomeric and fibrillar amyloid-*β*1–42 on astrocyte-mediated inflammation. *Neurobiology of Disease*.

[42] Fioravanzo L, Venturini M, Di Liddo R (2010). Involvement of rat hippocampal astrocytes in beta-amyloid-induced angiogenesis and neuroinflammation. *Current Alzheimer Research*.

[43] Abramov AY, Canevari L, Duchen MR (2003). Changes in intracellular calcium and glutathione in astrocytes as the primary mechanism of amyloid neurotoxicity. *The Journal of Neuroscience*.

[44] Allaman I, Gavillet M, Bélanger M (2010). Amyloid-*β* aggregates cause alterations of astrocytic metabolic phenotype: impact on neuronal viability. *The Journal of Neuroscience*.

[45] Gavillet M, Allaman I, Magistretti PJ (2008). Modulation of astrocytic metabolic phenotype by proinflammatory cytokines. *Glia*.

[46] Furman JL, Sama DM, Gant JC (2012). Targeting astrocytes ameliorates neurologic changes in a mouse model of Alzheimer's disease. *The Journal of Neuroscience*.

[47] von Bernhardi R, Eugenín J (2004). MicroGlial reactivity to *β*-amyloid is modulated by astrocytes and proinflammatory factors. *Brain Research*.

[48] Ramírez G, Toro R, Döbeli H, von Bernhardi R (2005). Protection of rat primary hippocampal cultures from A*β* cytotoxicity by pro-inflammatory molecules is mediated by astrocytes. *Neurobiology of Disease*.

[49] Alarcón R, Fuenzalida C, Santibáñez M, von Bernhardi R (2005). Expression of scavenger receptors in Glial cells: comparing the adhesion of astrocytes and microGlia from neonatal rats to surface-bound *β*-amyloid. *The Journal of Biological Chemistry*.

[50] Chang ZL (2010). Important aspects of Toll-like receptors, ligands and their signaling pathways. *Inflammation Research*.

[51] Ricevuti G, Mazzone A, Fossati G (1993). Assay of phagocytic cell functions. *Allergie et Immunologie*.

[52] Goldstein JL, Ho YK, Basu SK, Brown MS (1979). Binding site on macrophages that mediates uptake and degradation of acetylated low density lipoprotein, producing massive cholesterol deposition. *Proceedings of the National Academy of Sciences of the United States of America*.

[53] Hickman SE, Allison EK, El Khoury J (2008). MicroGlial dysfunction and defective *β*-amyloid clearance pathways in aging Alzheimer’s disease mice. *The Journal of Neuroscience*.

[54] Wilkinson K, El Khoury J (2012). MicroGlial scavenger receptors and their roles in the pathogenesis of Alzheimer's disease. *International Journal of Alzheimer's Disease*.

[55] Arai T, Rinninger F, Varban L (1999). Decreased selective uptake of high density lipoprotein cholesteryl esters in apolipoprotein E knock-out mice. *Proceedings of the National Academy of Sciences of the United States of America*.

[56] Krieger M, Kozarsky K (1999). Influence of the HDL receptor SR-BI on atherosclerosis. *Current Opinion in Lipidology*.

[57] Husemann J, Silverstein SC (2001). Expression of scavenger receptor class B, type I, by astrocytes and vascular smooth muscle cells in normal adult mouse and human brain and in Alzheimer’s disease brain. *American Journal of Pathology*.

[58] Baranova IN, Vishnyakova TG, Bocharov AV (2012). Class B scavenger receptor types I and II and CD36 mediate bacterial recognition and proinflammatory signaling induced by *Escherichia coli*, lipopolysaccharide, and cytosolic chaperonin 60. *The Journal of Immunology*.

[59] Buechler C, Ritter M, Quoc CD, Agildere A, Schmitz G (1999). Lipopolysaccharide inhibits the expression of the scavenger receptor Cla-1 in human monocytes and macrophages. *Biochemical and Biophysical Research Communications*.

[60] Guo L, Song Z, Li M (2009). Scavenger receptor BI protects against septic death through its role in modulating inflammatory response. *The Journal of Biological Chemistry*.

[61] Cai L, Wang Z, Meyer JM, Ji A, van der Westhuyzen DR (2012). Macrophage SR-BI regulates LPS-induced pro-inflammatory signaling in mice and isolated macrophages. *The Journal of Lipid Research*.

[62] Chang GHF, Barbaro NM, Pieper RO (2000). Phosphatidylserine-dependent phagocytosis of apoptotic glioma cells by normal human microGlia, astrocytes, and glioma cells. *Neuro-Oncology*.

[63] Husemann J, Loike JD, Kodama T, Silverstein SC (2001). Scavenger receptor class B type I (SR-BI) mediates adhesion of neonatal murine microGlia to fibrillar *β*-amyloid. *Journal of Neuroimmunology*.

[64] Thanopoulou K, Fragkouli A, Stylianopoulou F, Georgopoulos S (2010). Scavenger receptor class B type i (SR-BI) regulates perivascular macrophages and modifies amyloid pathology in an Alzheimer mouse model. *Proceedings of the National Academy of Sciences of the United States of America*.

[65] Febbraio M, Hajjar DP, Silverstein RL (2001). CD36: a class B scavenger receptor involved in angiogenesis, atherosclerosis, inflammation, and lipid metabolism. *The Journal of Clinical Investigation*.

[66] Hwang EH, Taki J, Yasue S (1998). Absent myocardial iodine-123-BMIPP uptake and platelet/monocyte CD36 deficiency. *Journal of Nuclear Medicine*.

[67] Savill J, Hogg N, Ren Y, Haslett C (1992). Thrombospondin cooperates with CD36 and the vitronectin receptor in macrophage recognition of neutrophils undergoing apoptosis. *The Journal of Clinical Investigation*.

[68] Barnwell JW, Asch AS, Nachman RL, Yamaya M, Aikawa M, Ingravallo P (1989). A human 88-kD membrane glycoprotein (CD36) functions in vitro as a receptor for a cytoadherence ligand on *Plasmodium falciparum*-infected erythrocytes. *The Journal of Clinical Investigation*.

[69] Bao Y, Qin L, Kim E (2012). CD36 is involved in astrocyte activation and astroGlial scar formation. *Journal of Cerebral Blood Flow and Metabolism*.

[70] Coraci IS, Husemann J, Berman JW (2002). CD36, a class B scavenger receptor, is expressed on microGlia in Alzheimer’s disease brains and can mediate production of reactive oxygen species in response to *β*-amyloid fibrils. *American Journal of Pathology*.

[71] Stuart LM, Bell SA, Stewart CR (2007). CD36 signals to the actin cytoskeleton and regulates microGlial migration via a p130Cas complex. *The Journal of Biological Chemistry*.

[72] Cho S, Park EM, Febbraio M (2005). The class B scavenger receptor CD36 mediates free radical production and tissue injury in cerebral ischemia. *The Journal of Neuroscience*.

[73] Kunz A, Abe T, Hochrainer K (2008). Nuclear factor-*κ*B activation and postischemic inflammation are suppressed in CD36-null mice after middle cerebral artery occlusion. *The Journal of Neuroscience*.

[74] Moore KJ, El Khoury J, Medeiros LA (2002). A CD36-initiated signaling cascade mediates inflammatory effects of *β*-amyloid. *The Journal of Biological Chemistry*.

[75] Bamberger ME, Harris ME, McDonald DR, Husemann J, Landreth GE (2003). A cell surface receptor complex for fibrillar *β*-amyloid mediates microGlial activation. *The Journal of Neuroscience*.

[76] El Khoury JB, Moore KJ, Means TK (2003). CD36 mediates the innate host response to *β*-amyloid. *Journal of Experimental Medicine*.

[77] Ricciarelli R, D’Abramo C, Zingg JM (2004). CD36 overexpression in human brain correlates with *β*-amyloid deposition but not with Alzheimer’s disease. *Free Radical Biology and Medicine*.

[78] Giunta M, Rigamonti AE, Scarpini E (2007). The leukocyte expression of CD36 is low in patients with Alzheimer’s disease and mild cognitive impairment. *Neurobiology of Aging*.

[79] Park L, Zhou J, Zhou P (2013). Innate immunity receptor CD36 promotes cerebral amyloid angiopathy. *Proceedings of the National Academy of Sciences of the United States of America*.

[80] Shimizu E, Kawahara K, Kajizono M, Sawada M, Nakayama H (2008). IL-4-induced selective clearance of oligomeric *β*-amyloid peptide 1–42 by rat primary type 2 microGlia. *The Journal of Immunology*.

[81] Jones RS, Minogue AM, Connor TJ, Lynch MA (2012). Amyloid-beta-induced astrocytic phagocytosis is mediated by CD36, CD47 and RAGE. *Journal of Neuroimmune Pharmacology*.

[82] Park L, Wang G, Zhou P (2011). Scavenger receptor CD36 is essential for the cerebrovascular oxidative stress and neurovascular dysfunction induced by amyloid-*β*. *Proceedings of the National Academy of Sciences of the United States of America*.

[83] Neeper M, Schmidt AM, Brett J (1992). Cloning and expression of a cell surface receptor for advanced glycosylation end products of proteins. *The Journal of Biological Chemistry*.

[84] Brett J, Schmidt AM, Yan SD (1993). Survey of the distribution of a newly characterized receptor for advanced glycation end products in tissues. *American Journal of Pathology*.

[85] Taguchi A, Blood DC, Del Toro G (2000). Blockade of RAGE-amphoterin signalling suppresses tumour growth and metastases. *Nature*.

[86] Yan SD, Schmidt AM, Anderson GM (1994). Enhanced cellular oxidant stress by the interaction of advanced glycation end products with their receptors/binding proteins. *The Journal of Biological Chemistry*.

[87] Andrassy M, Igwe J, Autschbach F (2006). Posttranslationally modified proteins as mediators of sustained intestinal inflammation. *American Journal of Pathology*.

[88] Giri R, Shen Y, Stins M (2000). *β*-Amyloid-induced migration of monocytes across human brain endothelial cells involves RAGE and PECAM-1. *American Journal of Physiology*.

[89] Arancio O, Zhang HP, Chen X (2004). RAGE potentiates A*β*-induced perturbation of neuronal function in transgenic mice. *The EMBO Journal*.

[90] OriGlia N, Bonadonna C, Rosellini A (2010). MicroGlial receptor for advanced glycation end product-dependent signal pathway drives *β*-amyloid-induced synaptic depression and long-term depression impairment in entorhinal cortex. *The Journal of Neuroscience*.

[91] Vodopivec I, Galichet A, Knobloch M, Bierhaus A, Heizmann CW, Nitsch RM (2009). RAGE does not affect amyloid pathology in transgenic arcA*β* mice. *Neurodegenerative Diseases*.

[92] Kodama T, Freeman M, Rohrer L, Zabrecky J, Matsudaira P, Krieger M (1990). Type I macrophage scavenger receptor contains *α*-helical and collagen-like coiled coils. *Nature*.

[93] Rohrer L, Freeman M, Kodama T, Penman M, Krieger M (1990). Coiled-coil fibrous domains mediate ligand binding by macrophage scavenger receptor type II. *Nature*.

[94] Matsumoto A, Naito M, Itakura H (1990). Human macrophage scavenger receptors: primary structure, expression, and localization in atherosclerotic lesions. *Proceedings of the National Academy of Sciences of the United States of America*.

[95] El Khoury J, Thomas CA, Loike JD, Hickman SE, Cao L, Silverstein SC (1994). Macrophages adhere to glucose-modified basement membrane collagen IV via their scavenger receptors. *The Journal of Biological Chemistry*.

[96] Hampton RY, Golenbock DT, Penman M, Krieger M, Raetz CRH (1991). Recognition and plasma clearance of endotoxin by scavenger receptors. *Nature*.

[97] Thomas CA, Li Y, Kodama T, Suzuki H, Silverstein SC, El Khoury J (2000). Protection from lethal gram-positive infection by macrophage scavenger receptor-dependent phagocytosis. *Journal of Experimental Medicine*.

[98] Suzuki H, Kurihara Y, Takeya M (1997). A role for macrophage scavenger receptors in atherosclerosis and susceptibility to infection. *Nature*.

[99] Kobayashi Y, Miyaji C, Watanabe H (2000). Role of macrophage scavenger receptor in endotoxin shock. *Journal of Pathology*.

[100] Yi H, Yu X, Gao P (2009). Pattern recognition scavenger receptor SRA/CD204 down-regulates Toll-like receptor 4 signaling-dependent CD8 T-cell activation. *Blood*.

[101] Chen Y, Wermeling F, Sundqvist J (2010). A regulatory role for macrophage class A scavenger receptors in TLR4-mediated LPS responses. *European Journal of Immunology*.

[102] Ohnishi K, Komohara Y, Fujiwara Y (2011). Suppression of TLR4-mediated inflammatory response by macrophage class A scavenger receptor (CD204). *Biochemical and Biophysical Research Communications*.

[103] Yu X, Yi H, Guo C (2011). Pattern recognition scavenger receptor CD204 attenuates toll-like receptor 4-induced NF-*κ*B activation by directly inhibiting ubiquitination of Tumor Necrosis Factor (TNF) receptor-associated factor 6. *The Journal of Biological Chemistry*.

[104] Yu H, Ha T, Liu L (2012). Scavenger receptor A, (SR-A) is required for LPS-induced TLR4 mediated NF-kappaB activation in macrophages. *Biochimica et Biophysica Acta*.

[105] Fadok VA, Voelker DR, Campbell PA, Cohen JJ, Bratton DL, Henson PM (1992). Exposure of phosphatidylserine on the surface of apoptotic lymphocytes triggers specific recognition and removal by macrophages. *The Journal of Immunology*.

[106] Platt N, Suzuki H, Kurihara Y, Kodama T, Gordon S (1996). Role for the class A macrophage scavenger receptor in the phagocytosis of apoptotic thymocytes in vitro. *Proceedings of the National Academy of Sciences of the United States of America*.

[107] Hu Y, Zhang H, Lu Y (2012). Class A scavenger receptor attenuates myocardial infarction-induced cardiomyocyte necrosis through suppressing M1 macrophage subset polarization. *Basic Research in Cardiology*.

[108] Nikolic D, Calderon L, Du L, Post SR (2011). SR-A ligand and M-CSF dynamically regulate SR-A expression and function in primary macrophages via p38 MAPK activation. *BMC Immunology*.

[109] Cholewa J, Nikolic D, Post SR (2010). Regulation of class a scavenger receptor-mediated cell adhesion and surface localization by PI3K: identification of a regulatory cytoplasmic motif. *Journal of Leukocyte Biology*.

[110] Bell MD, Lopez-Gonzalez R, Lawson L (1994). Upregulation of the macrophage scavenger receptor in response to different forms of injury in the CNS. *Journal of Neurocytology*.

[111] Christie RH, Freeman M, Hyman BT (1996). Expression of the macrophage scavenger receptor, a multifunctional lipoprotein receptor, in microGlia associated with senile plaques in Alzheimer’s disease. *American Journal of Pathology*.

[112] Lucarelli M, Gennarelli M, Cardelli P (1997). Expression of receptors for native and chemically modified low-density lipoproteins in brain microvessels. *FEBS Letters*.

[113] Grewal RP, Yoshida T, Finch CE, Morgan TE (1997). Scavenger receptor mRNAs in rat brain microGlia are induced by kainic acid lesioning and by cytokines. *NeuroReport*.

[114] Honda M, Akiyama H, Yamada Y (1998). Immunohistochemical evidence for a macrophage scavenger receptor in Mato cells and reactive microGlia of ischemia and Alzheimer’s disease. *Biochemical and Biophysical Research Communications*.

[115] Godoy B, Murgas P, Tichauer J, von Bernhardi R (2012). Scavenger receptor class A ligands induce secretion of IL1beta and exert a modulatory effect on the inflammatory activation of astrocytes in culture. *Journal of Neuroimmunology*.

[116] Lu C, Hua F, Liu L (2010). Scavenger receptor class-A has a central role in cerebral ischemia-reperfusion injury. *Journal of Cerebral Blood Flow and Metabolism*.

[117] Xu Y, Qian L, Zong G (2012). Class A scavenger receptor promotes cerebral ischemic injury by pivoting microGlia/macrophage polarization. *Neuroscience*.

[118] Ren D, Wang X, Ha T (2013). SR-A deficiency reduces myocardial ischemia/reperfusion injury, involvement of increased microRNA-125b expression in macrophages. *Biochimica et Biophysica Acta*.

[119] Bornemann KD, Wiederhold KH, Pauli C (2001). A*β*-induced inflammatory processes in microGlia cells of APP23 transgenic mice. *American Journal of Pathology*.

[120] El Khoury J, Hickman SE, Thomas CA, Cao L, Silverstein SC, Loike JD (1996). Scavenger receptor-mediated adhesion of microGlia to *β*-amyloid fibrils. *Nature*.

[121] Prior R, Wihl G, Urmoneit B (2000). Apolipoprotein E, smooth muscle cells and the pathogenesis of cerebral amyloid angiopathy: the potential pole of impaired cerebrovascular A*β* clearance. *Annals of the New York Academy of Sciences*.

[122] Chung H, Brazil MI, Irizarry MC, Hyman BT, Maxfield FR (2001). Uptake of fibrillar *β*-amyloid by microGlia isolated from MSR-A (type I and type II) knockout mice. *NeuroReport*.

[123] Yang CN, Shiao YJ, Shie FS (2011). Mechanism mediating oligomeric Abeta clearance by naive primary microGlia. *Neurobiology of Disease*.

[124] Elomaa O, Kangas M, Sahlberg C (1995). Cloning of a novel bacteria-binding receptor structurally related to scavenger receptors and expressed in a subset of macrophages. *Cell*.

[125] van der Laan LJW, Kangas M, Döpp EA (1997). Macrophage scavenger receptor marco: in vitro and in vivo regulation and involvement in the anti-bacterial host defense. *Immunology Letters*.

[126] van der Laan LJW, Döpp EA, Haworth R (1999). Regulation and functional involvement of macrophage scavenger receptor MARCO in clearance of bacteria in vivo. *The Journal of Immunology*.

[127] Pikkarainen T, Brännström A, Tryggvason K (1999). Expression of macrophage MARCO receptor induces formation of dendritic plasma membrane processes. *The Journal of Biological Chemistry*.

[128] Granucci F, Petralia F, Urbano M (2003). The scavenger receptor MARCO mediates cytoskeleton rearrangements in dendritic cells and microGlia. *Blood*.

[129] Martinez-Pomares L, Platt N, Mcknight AJ, Da Silva RP, Gordon S (1996). Macrophage membrane molecules: markers of tissue differentiation and heterogeneity. *Immunobiology*.

[130] Argiles A, Mourad G, Kerr PG, Garcia M, Collins B, Demaille JG (1994). Cells surrounding haemodialysis-associated amyloid deposits are mainly macrophages. *Nephrology Dialysis Transplantation*.

[131] Yoshida H, Kondratenko N, Green S, Steinberg D, Quehenberger O (1998). Identification of the lectin-like receptor for oxidized low-density lipoprotein in human macrophages and its potential role as a scavenger receptor. *Biochemical Journal*.

[132] Luedecking-Zimmer E, DeKosky ST, Chen Q, Barmada MM, Kamboh MI (2002). Investigation of oxidized LDL-receptor 1 (ORL1) as the candidate gene for Alzheimer’s disease on chromosome 12. *Human Genetics*.

[133] Shi J, Tian J, Pritchard A (2006). A 3'-UTR polymorphism in the oxidized LDL receptor 1 gene increases A*β*40 load as cerebral amyloid angiopathy in Alzheimer’s disease. *Acta Neuropathologica*.

[134] Suzuki E, Nakayama M (2007). The mammalian Ced-1 ortholog MEGF10/KIAA1780 displays a novel adhesion pattern. *Experimental Cell Research*.

[135] Singh TD, Park SY, Bae JS (2010). MEGF10 functions as a receptor for the uptake of amyloid-*β*. *FEBS Letters*.

